# Analysis of Eyring-Powell Fluid in Helical Screw Rheometer

**DOI:** 10.1155/2014/143968

**Published:** 2014-02-24

**Authors:** A. M. Siddiqui, T. Haroon, M. Zeb

**Affiliations:** ^1^Department of Mathematics, York Campus, Pennsylvania State University, York, PA 17403, USA; ^2^Department of Mathematics, COMSATS Institute of Information Technology, 44000 Islamabad, Pakistan; ^3^Department of Mathematics, COMSATS Institute of Information Technology, 43600 Attock, Pakistan

## Abstract

This paper aims to study the flow of an incompressible, isothermal Eyring-Powell fluid in a helical screw rheometer. The complicated geometry of the helical screw rheometer is simplified by “unwrapping or flattening” the channel, lands, and the outside rotating barrel, assuming the width of the channel is larger as compared to the depth. The developed second order nonlinear differential equations are solved by using Adomian decomposition method. Analytical expressions are obtained for the velocity profiles, shear stresses, shear at wall, force exerted on fluid, volume flow rates, and average velocity. The effect of non-Newtonian parameters, pressure gradients, and flight angle on the velocity profiles is noticed with the help of graphical representation. The observation confirmed the vital role of involved parameters during the extrusion process.

## 1. Introduction

The study of the rheological characteristics of different fluids is essential in the process of processing to obtain the desired quality and shape of the products. During processing, noticeable physical and chemical changes can occur [1]. For measuring the rheological properties of fluids in industries, mostly in the food industry, the available instruments are different types of viscometers. All these viscometers have their own advantages and limitations, they do not measure the fundamental physical parameters absolutely and they are empirical in nature [2, 3]. Kraynik et al. [4] have developed an instrument alternative to viscometers, called helical screw rheometer (HSR), for use in coal liquefaction processing that could characterize fluid suspensions accurately and consistently.

In order to optimize the processing and improve the quality of production, the literature on the classical extrusion theory contains the work of Carley et al. [5], Mohr and Mallouk [6], Booy [7], and Bird et al. [8].

Tamura et al. [9] have tried successfully the preceding analysis in the geometry of helical screw rheometer for Newtonian fluid and Power law fluid. In recent years, the study of non-Newtonian fluids have attracted many researchers. This is mostly due to their wide use in the food industry, chemical process industry, construction engineering, power engineering, petroleum production, and commercial and technological applications. Examples of non-Newtonian fluids are industrial materials, such as polymer melts, paints, gels, rubbers, soaps, inks, oils, concrete, ketchup, pastes, suspensions, slurries, and biological liquids such as blood and foodstuffs. The rheological knowledge of such fluids is of special importance owing to its application to many industrial problems. Considerable efforts have been made towards understanding their flows. Different models are available to characterize the non-Newtonian behavior of fluids out of which the fluid based on Eyring-Powell model is chosen for simplicity in the present work.

The basic governing equations for non-Newtonian fluids motion are highly nonlinear differential equations having no general solution, and only a limited number of exact solutions have been established for particular problems. Therefore, these problems should be treated by using some numerical or analytical methods. The analytical study of such type of nonlinear problems is important not only because of their technological significance, but also due to the interesting mathematical features presented by the governing differential equations of the flow. Apart from numerical methods, several analytical techniques such as the regular perturbation technique [10], the homotopy analysis method (HAM), the homotopy perturbation method (HPM) [11, 12], the variational iteration method (VIM) [13, 14], and the Adomian decomposition method (ADM) are mostly in use to overcome nonlinearity and get solutions. In this paper, we aim to apply the iterative technique ADM, which was introduced and developed by George Adomian and well addressed in the literature. ADM has recently received ample attention in the area of series solutions. A considerable amount of research work has been invested in the application of this method to a wide class of linear, nonlinear, partial differential equations, and integral equations [15–23].

This work considered the steady flow of an incompressible, isothermal, and homogeneous Eyring-Powell fluid in helical screw rheometer (HSR). Using Adomian decomposition method (ADM), analytical solutions are obtained for the governing equations in the geometry under consideration. Expressions of the velocity profiles, shear stresses, shear stresses at wall, forces exerted on fluid, volume flow rates, and average velocity are also calculated. The effect of involved dimensionless parameters on flow profiles are investigated through graphs and are discussed.

The paper is organized as follows.  Section 2 contains the basic equations governing the motion of the fluid. In Section 3 the problem under consideration is formulated.  Section 4 is devoted to the analytical solutions of the flow profiles, shear stresses, shear stresses at wall, forces exerted on fluid, volume flow rates, and average velocity.  Section 5 is related to the discussion about the effect of the involved parameters on the motion of the fluid. Appropriate conclusions are drawn in Section 6.

## 2. Basic Equations

The equations of conservation of mass and momentum for an incompressible fluid are

(1) $$\begin{array}{c} \mathrm{div}V=0, \end{array}$$

(2) $$\begin{array}{c} \rho \frac{DV}{Dt}=\rho f+\mathrm{div}T, \end{array}$$

where *ρ* is the constant fluid density, *D*/*Dt* denotes the material time derivative, **V** is the velocity field, **f** is the body force per unit mass, and **T** is the Cauchy stress tensor expressed as

(3) $$\begin{array}{c} T=-PI+S, \end{array}$$

where *P* denotes the dynamic pressure, **I** the unit tensor, and **S** denotes the extra stress tensor.

The constitutive equation for Eyring-Powell fluid is given by [24] as

(4) $$\begin{array}{c} S=\mu A_{1}+\left[\frac{\left(1/ℬ\right)\sinh^{-1}\left(\left(1/𝒞\right)\left|A_{1}\right|\right)}{\left|A_{1}\right|}\right]A_{1}, \end{array}$$

where *μ* is viscosity, *𝒞*, *ℬ* are material constants with dimensions second^−1^ and Pascal^−1^, respectively, $|A_{1}|=\sqrt{\left(1/2)\mathrm{tr}(A_{1}^{2}\right)}$ , and **A**_1_ is the Rivlin-Ericksen tensor defined as

(5) $$\begin{array}{c} A_{1}=\left(\nabla V\right)+{\left(\nabla V\right)}^{T}, \end{array}$$

where ∇**V** is the velocity gradient.

## 3. Problem Formulation

**Figure 1:**
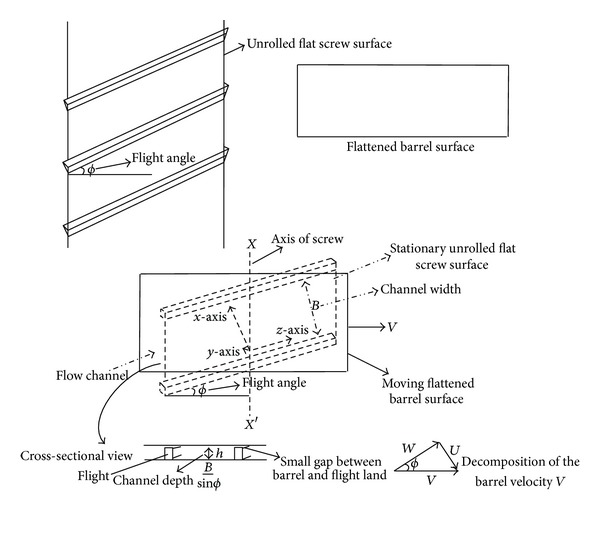
The geometry of the “unwrapped” screw channel and barrel surface.

Consider the steady flow of an isothermal, incompressible, and homogeneous Eyring-Powell fluid in helical screw rheometer (HSR). The complicated geometry of HSR is simplified in such a way that the curvature of the screw channel is ignored, unrolled, and laid out on a flat surface. The barrel surface is also flattened. Assume that the screw surface, the lower plate, is stationary and the barrel surface, the upper plate, is moving across the top of the channel with velocity *V* at an angle *ϕ* to the direction of the channel (Figure 1). The phenomenon is same as the barrel held stationary and the screw rotates. The geometry is approximated as a shallow infinite channel by assuming that the width *B* of the channel is large compared with the depth *h*; edge effects in the fluid at the land are ignored. The coordinate axes are positioned in such a way that the *x*-axis is perpendicular to the flight walls, *y*-axis is normal to the barrel surface, and *z*-axis is in down channel direction. The liquid wets all the surfaces and moves by the shear stresses produced by the relative movement of the barrel and channel. No leakage of the fluid occurs across the flights. For simplicity, the velocity of the barrel relative to the channel is decomposed into two components (see Figure 1): *U* along *x*-axis and *W* along *z*-axis [6]. The associated boundary conditions can be taken as seen below (Figure 1)

(6) $$\begin{array}{c} u=0,\;w=0,\;\text{at}\;y=0,\\ u=U,\;w=W,\;\text{at}\;y=h, \end{array}$$

where

(7) $$\begin{array}{c} U=-V\;\sin\phi ,\;\;W=V\;\cos\phi . \end{array}$$

The geometry of the problem suggests that the velocity profile **V** and extra stress tensor **S** are

(8) $$\begin{array}{c} V=\left[u\left(y\right),0,w\left(y\right)\right],\;\;S=S\left(y\right). \end{array}$$

Using (8) in (5) and (4), we get the nonzero components of the extra stress tensor given as

(9) $$\begin{array}{c} S_{xy}=S_{yx}=\mu \frac{du}{dy}+\frac{1}{ℬ}\sinh^{-1}\left(\frac{1}{𝒞}\frac{du}{dy}\right),\\ S_{yz}=S_{zy}=\mu \frac{dw}{dy}+\frac{1}{ℬ}\sinh^{-1}\left(\frac{1}{𝒞}\frac{dw}{dy}\right). \end{array}$$

Using (8), (1) is identically satisfied and the substitution of the velocity profile of (8) and (9) in (2), in the absence of body forces results in

(10) $$\begin{array}{c} \frac{\partial P}{\partial x}=\mu \frac{d^{2}u}{dy^{2}}+\frac{1}{ℬ}\frac{d}{dy}\left[\sinh^{-1}\left(\frac{1}{𝒞}\frac{du}{dy}\right)\right], \end{array}$$

(11) $$\begin{array}{c} \frac{\partial P}{\partial y}=0, \end{array}$$

(12) $$\begin{array}{c} \frac{\partial P}{\partial z}=\mu \frac{d^{2}w}{dy^{2}}+\frac{1}{ℬ}\frac{d}{dy}\left[\sinh^{-1}\left(\frac{1}{𝒞}\frac{dw}{dy}\right)\right]. \end{array}$$

Equation (11) shows that *P* = *P*(*x*, *z*), since the right sides of (10) and (12) are functions of *y* alone and *P* ≠ *P*(*y*); this implies ∂*P*/∂*x* = constant and ∂*P*/∂*z* = constant. Maclaurin series expansion of the inverse sine hyperbolic function in (10) and (12), when neglecting higher powers as |(1/*𝒞*)(*du*/*dy*)| ≪ 1, is

(13) $$\begin{array}{c} \frac{\partial P}{\partial x}=\mu \frac{d^{2}u}{dy^{2}}+\frac{1}{ℬ}\frac{d}{dy}\left[\frac{1}{𝒞}\frac{du}{dy}-\frac{1}{6}{\left(\frac{1}{𝒞}\frac{du}{dy}\right)}^{3}\right],\\ \frac{\partial P}{\partial z}=\mu \frac{d^{2}w}{dy^{2}}+\frac{1}{ℬ}\frac{d}{dy}\left[\frac{1}{𝒞}\frac{dw}{dy}-\frac{1}{6}{\left(\frac{1}{𝒞}\frac{dw}{dy}\right)}^{3}\right]. \end{array}$$

By simplifying (13), we obtained

(14) d2udy2=(𝒞ℬμ𝒞ℬ+1)∂P∂x+(12𝒞2(μ𝒞ℬ+1))(dudy)2d2udy2,d2wdy2=(𝒞ℬμ𝒞ℬ+1)∂P∂z+(12𝒞2(μ𝒞ℬ+1))(dwdy)2d2wdy2.

Introducing the dimensionless parameters

(15) $$\begin{array}{c} x^{*}=\frac{x}{h},\;\;y^{*}=\frac{y}{h},\;\;z^{*}=\frac{z}{h},\\ u^{*}=\frac{u}{W},\;\;w^{*}=\frac{w}{W},\;\;P^{*}=\frac{P}{\mu W/h}, \end{array}$$

in (6) and (14), we get

(16) $$\begin{array}{c} \frac{d^{2}u^{*}}{dy^{*2}}=\alpha^{*}\frac{\partial P^{*}}{\partial x^{*}}+\beta^{*}{\left(\frac{du^{*}}{dy^{*}}\right)}^{2}\frac{d^{2}u^{*}}{dy^{*2}},\\ \frac{d^{2}w^{*}}{dy^{*2}}=\alpha^{*}\frac{\partial P^{*}}{\partial z^{*}}+\beta^{*}{\left(\frac{dw^{*}}{dy^{*}}\right)}^{2}\frac{d^{2}w^{*}}{dy^{*2}}, \end{array}$$

where α* = ((*μ𝒞ℬ*)/(*μ𝒞ℬ* + 1)) and β* = (*W* ^2^/(2*𝒞* ^2^ *h* ^2^(*μ𝒞ℬ* + 1))) are dimensionless non-Newtonian parameters. Equation (6), in dimensionless form reduces to

(17) $$\begin{array}{c} u^{*}=0,\;\text{at}\;y^{*}=0,\;\\ u^{*}=-\tan\phi ,\;\text{at}\;y^{*}=1,\\ w^{*}=0,\;\text{at}\;y^{*}=0,\\ w^{*}=1,\;\text{at}{\;y}^{*}=1. \end{array}$$

Dropping “∗” onward (16)-(17), give

(18) $$\begin{array}{c} \frac{d^{2}u}{dy^{2}}=\alpha \frac{\partial P}{\partial x}+\beta {\left(\frac{du}{dy}\right)}^{2}\frac{d^{2}u}{dy^{2}}, \end{array}$$

(19) $$\begin{array}{c} \frac{d^{2}w}{dy^{2}}=\alpha \frac{\partial P}{\partial z}+\beta {\left(\frac{dw}{dy}\right)}^{2}\frac{d^{2}w}{dy^{2}}, \end{array}$$

(20) $$\begin{array}{c} u=0,\;\text{at}\;y=0, \end{array}$$

Equations (18) and (19) are second order nonlinear ordinary differential equations, with boundary conditions (20); the exact solutions seem to be difficult. In the following section we use the Adomian decomposition method to obtain the approximate solutions.

## 4. Solution of the Problem

The Adomian decomposition method (ADM) [15–23] describes that the operator form (18) and (19) can be written as in

(21) $$\begin{array}{c} L_{yy}\left(u\right)=\alpha \frac{\partial P}{\partial x}+\beta {\left(\frac{du}{dy}\right)}^{2}\frac{d^{2}u}{dy^{2}},\\ L_{yy}\left(w\right)=\alpha \frac{\partial P}{\partial z}+\beta {\left(\frac{dw}{dy}\right)}^{2}\frac{d^{2}w}{dy^{2}}, \end{array}$$

where *L*_*yy*_ = *d* ^2^/*dy* ^2^ is the differential operator taken as the highest order derivative to avoid difficult integrations. Since *L*_*yy*_ is invertible, this implies that *L*_*yy*_^−1^ = ∬(∗)*dy* *dy* exists.

On applying *L*_*yy*_^−1^ to both sides of (21), we obtained

(22) $$\begin{array}{c} u=C_{1}+C_{2}y+L_{yy}^{-1}\left(\alpha \frac{\partial P}{\partial x}\right)+\beta L_{yy}^{-1}\left[{\left(\frac{du}{dy}\right)}^{2}\frac{d^{2}u}{dy^{2}}\right],\\ w=C_{3}+C_{4}y+L_{yy}^{-1}\left(\alpha \frac{\partial P}{\partial z}\right)+\beta L_{yy}^{-1}\left[{\left(\frac{dw}{dy}\right)}^{2}\frac{d^{2}w}{dy^{2}}\right], \end{array}$$

where *C*_1_, *C*_2_, *C*_3_, and *C*_4_ are arbitrary constants of integration and can be determined by using boundary conditions. According to the procedure of ADM, *u* and *w* can be written in component form as

(23) $$\begin{array}{c} u=\sum_{n=0}^{\infty }u_{n},\\ w=\sum_{n=0}^{\infty }w_{n}. \end{array}$$

Using (23) in (22) results in

(24) u=C1+C2y+Lyy−1(α∂P∂x)+βLyy−1[(ddy∑n=0∞un)2(d2dy2∑n=0∞un)],w=C3+C4y+Lyy−1(α∂P∂z)+βLyy−1[(ddy∑n=0∞wn)2(d2dy2∑n=0∞wn)].

Since the nonlinear terms can be explored in the form of the Adomian polynomials, say Λ_*n*_ and Γ_*n*_,

(25) ∑n=0∞Λn=(ddy∑n=0∞un)2(d2dy2∑n=0∞un),∑n=0∞Γn=(ddy∑n=0∞wn)2(d2dy2∑n=0∞wn),

which yield (24), in the form

(26) u=C1+C2y+Lyy−1(α∂P∂x)+βLyy−1(∑n=0∞Λn),

(27) w=C3+C4y+Lyy−1(α∂P∂z)+βLyy−1(∑n=0∞Γn),

and the boundary conditions (20) will take the form

(28) $$\begin{array}{c} \sum_{n=0}^{\infty }u_{n}=0,\;\text{at}\;y=0,\\ \sum_{n=0}^{\infty }u_{n}=-\tan\phi ,\;\text{at}\;y=1, \end{array}$$

(29) $$\begin{array}{c} \sum_{n=0}^{\infty }w_{n}=0,\;\text{at}\;y=0,\\ \sum_{n=0}^{\infty }w_{n}=1,\;\text{at}\;y=1. \end{array}$$

From the recursive relation in (26)–(29), we can identify the zeroth order problems as

(30) $$\begin{array}{c} u_{0}=C_{1}+C_{2}y+L_{yy}^{-1}\left(\alpha \frac{\partial P}{\partial x}\right), \end{array}$$

(31) $$\begin{array}{c} w_{0}=C_{3}+C_{4}y+L_{yy}^{-1}\left(\alpha \frac{\partial P}{\partial z}\right), \end{array}$$

with boundary conditions

(32) $$\begin{array}{c} u_{0}=0,\;\text{at}\;y=0,\\ u_{0}=-\tan\phi ,\;\text{at}\;y=1, \end{array}$$

(33) $$\begin{array}{c} w_{0}=0,\;\text{at}\;y=0,\\ w_{0}=1,\;\;\text{at}\;y=1. \end{array}$$

The remaining order problems are in the following form:

(34) $$\begin{array}{c} u_{j+1}=\beta L_{yy}^{-1}\left(\Lambda_{j}\right),\;j\geq 0, \end{array}$$

(35) $$\begin{array}{c} w_{j+1}=\beta L_{yy}^{-1}\left(\Gamma_{j}\right),\;j\geq 0, \end{array}$$

with the boundary conditions

(36) $$\begin{array}{c} \sum_{j=1}^{\infty }u_{j}=0,\;\text{at}\;y=0,\\ \sum_{j=1}^{\infty }u_{j}=0,\;\text{at}\;y=1, \end{array}$$

(37) $$\begin{array}{c} \sum_{j=1}^{\infty }w_{j}=0,\;\text{at}\;y=0,\\ \sum_{j=1}^{\infty }w_{j}=0,\;\text{at}\;y=1. \end{array}$$

From (25) we can calculate the components of the Adomian polynomials, say Λ_*n*_ and Γ_*n*_, as

(38) $$\begin{array}{c} \Lambda_{0}={\left(\frac{du_{0}}{dy}\right)}^{2}\frac{d^{2}u_{0}}{dy^{2}}, \end{array}$$

(39) $$\begin{array}{c} \Lambda_{1}={\left(\frac{du_{0}}{dy}\right)}^{2}\frac{d^{2}u_{1}}{dy^{2}}+2\frac{du_{0}}{dy}\frac{du_{1}}{dy}\frac{d^{2}u_{0}}{dy^{2}}, \end{array}$$

(40) Λ2=(du1dy)2d2u0dy2+2du0dydu1dyd2u1dy2+2du0dydu2dyd2u0dy2+(du0dy)2d2u2dy2,

(41) $$\begin{array}{c} \Gamma_{0}={\left(\frac{dw_{0}}{dy}\right)}^{2}\frac{d^{2}w_{0}}{dy^{2}}, \end{array}$$

(42) $$\begin{array}{c} \Gamma_{1}={\left(\frac{dw_{0}}{dy}\right)}^{2}\frac{d^{2}w_{1}}{dy^{2}}+2\frac{dw_{0}}{dy}\frac{dw_{1}}{dy}\frac{d^{2}w_{0}}{dy^{2}}, \end{array}$$

(43) Γ2=(dw1dy)2d2w0dy2+2dw0dydw1dyd2w1dy2+2dw0dydw2dyd2w0dy2+(dw0dy)2d2w2dy2;

the remaining components of the Adomian polynomials can be generated easily.

The ADM solutions to (26) and (27) with the boundary conditions (28) and (29) will be the sum of all order solutions; that is,

(44) $$\begin{array}{c} u=\sum_{n=1}^{\infty }u_{n},\\ w=\sum_{n=1}^{\infty }w_{n}. \end{array}$$

### 4.1. Zeroth Order Solution

Zeroth order solutions of (18)–(20) can be calculated from the relations given in (30)–(33), which are

(45) $$\begin{array}{c} u_{0}=-y\tan\phi +\frac{\alpha }{2}\frac{\partial P}{\partial x}\left(y^{2}-y\right), \end{array}$$

(46) $$\begin{array}{c} w_{0}=y+\frac{\alpha }{2}\frac{\partial P}{\partial z}\left(y^{2}-y\right); \end{array}$$

equations (45) and (46) give the solutions for the linearly viscous fluid by assuming *α* = 1/*μ*.

### 4.2. First Order Solution

Equations (34)–(37) give the first order problems as

(47) $$\begin{array}{c} u_{1}=\beta L_{yy}^{-1}\left(\Lambda_{0}\right), \end{array}$$

(48) $$\begin{array}{c} w_{1}=\beta L_{yy}^{-1}\left(\Gamma_{0}\right), \end{array}$$

with the boundary conditions

(49) $$\begin{array}{c} u_{1}=0,\;\text{at}\;y=0,\\ u_{1}=0,\;\text{at}\;y=1, \end{array}$$

(50) $$\begin{array}{c} w_{1}=0,\;\text{at}\;y=0,\\ w_{1}=0,\;\text{at}\;y=1. \end{array}$$

Using (38) and (41) in (47)–(50), we get

(51) $$\begin{array}{c} u_{1}=\beta \left(\frac{\epsilon_{0}}{2}\left(y^{2}-y\right)+\frac{\epsilon_{1}}{6}\left(y^{3}-y\right)+\frac{\epsilon_{2}}{12}\left(y^{4}-y\right)\right), \end{array}$$

(52) $$\begin{array}{c} w_{1}=\beta \left(\frac{\sigma_{0}}{2}\left(y^{2}-y\right)+\frac{\sigma_{1}}{6}\left(y^{3}-y\right)+\frac{\sigma_{2}}{12}\left(y^{4}-y\right)\right), \end{array}$$

where

(53) ϵ0=αtan2ϕ∂P∂x+α2tanϕ(∂P∂x)2+14α3(∂P∂x)3,ϵ1=−2α2tanϕ(∂P∂x)2−α3(∂P∂x)3,ϵ2=α3(∂P∂x)3σ0=α∂P∂z−α2(∂P∂z)2+14α3(∂P∂z)3,σ1=2α2(∂P∂z)2−α3(∂P∂z)3,σ2=α3(∂P∂z)3

are constant coefficients.

### 4.3. Second Order Solution

Equations (34)–(37) give the second order problems as

(54) $$\begin{array}{c} u_{2}=\beta L_{yy}^{-1}\left(\Lambda_{1}\right), \end{array}$$

(55) $$\begin{array}{c} w_{2}=\beta L_{yy}^{-1}\left(\Gamma_{1}\right), \end{array}$$

with the boundary conditions

(56) $$\begin{array}{c} u_{2}=0,\;\text{at}\;y=0,\\ u_{2}=0,\;\text{at}\;y=1, \end{array}$$

(57) $$\begin{array}{c} w_{2}=0,\;\text{at}\;y=0,\\ w_{2}=0,\;\text{at}\;y=1. \end{array}$$

Using (39) and (42) in (54)–(57), we obtained

(58) u2=β2(ϵ32(y2−y)+ϵ46(y3−y)  +ϵ512(y4−y)+ϵ620(y5−y)+ϵ730(y6−y)),

(59) w2=β2(σ32(y2−y)+σ46(y3−y)  +σ512(y4−y)+σ620(y5−y)+σ730(y6−y)),

where

(60) ϵ3=αtan4ϕ∂P∂x+3α2tan3ϕ(∂P∂x)2+73α3tan2ϕ(∂P∂x)3+34α4tanϕ(∂P∂x)4+548α5(∂P∂x)5,ϵ4=−6α2tan3ϕ(∂P∂x)2−10α3tan2ϕ(∂P∂x)3−296α4tanϕ(∂P∂x)4−56α5(∂P∂x)5,ϵ5=10α3tan2ϕ(∂P∂x)3+10α4tanϕ(∂P∂x)4+52α5(∂P∂x)5,ϵ6=−203α4tanϕ(∂P∂x)4−103α5(∂P∂x)5,ϵ7=53α5(∂P∂x)5,σ3=α∂P∂z−3α2(∂P∂z)2+73α3(∂P∂z)3−34α4(∂P∂z)4+548α5(∂P∂z)5,σ4=6α2(∂P∂z)2−10α3(∂P∂z)3+296α4(∂P∂z)4−56α5(∂P∂z)5,σ5=10α3(∂P∂z)3−10α4(∂P∂z)4+52α5(∂P∂z)5,σ6=203α4(∂P∂z)4−103α5(∂P∂z)5,σ7=53α5(∂P∂z)5

are constant coefficients in (58) and (59).

### 4.4. Third Order Solution

Equations (34)–(37) give the third order problems as

(61) $$\begin{array}{c} u_{3}=\beta L_{yy}^{-1}\left(\Lambda_{2}\right), \end{array}$$

(62) $$\begin{array}{c} w_{3}=\beta L_{yy}^{-1}\left(\Gamma_{2}\right), \end{array}$$

with the boundary conditions

(63) $$\begin{array}{c} u_{3}=0,\;\text{at}\;y=0,\\ u_{3}=0,\;\text{at}\;y=1, \end{array}$$

(64) $$\begin{array}{c} w_{3}=0,\;\text{at}\;y=0,\\ w_{3}=0,\;\text{at}\;y=1. \end{array}$$

Using (40) and (43) in (61)–(64), we get

(65) u3=β3(ϵ82(y2−y)+ϵ96(y3−y)+ϵ1012(y4−y)  +ϵ1120(y5−y)+ϵ1230(y6−y)  +ϵ1342(y7−y)+ϵ1456(y8−y)),

(66) w3=β3(σ82(y2−y)+σ96(y3−y)+σ1012(y4−y)  +σ1120(y5−y)+σ1230(y6−y)  +σ1342(y7−y)+σ1456(y8−y)),

where

(67) ϵ8=αtan6ϕ∂P∂x+6α2tan5ϕ(∂P∂x)2+556α3tan4ϕ(∂P∂x)3+132α4tan3ϕ(∂P∂x)4+11948α5tan2ϕ(∂P∂x)5+12α6tanϕ(∂P∂x)6+7144α7(∂P∂x)7,ϵ9=−12α2tan5ϕ(∂P∂x)2−40α3tan4ϕ(∂P∂x)3−43α4tan3ϕ(∂P∂x)4−653α5tan2ϕ(∂P∂x)5−9718α6tanϕ(∂P∂x)6−712α7(∂P∂x)7,ϵ10=40α3tan4ϕ(∂P∂x)3+90α4tan3ϕ(∂P∂x)4+2053α5tan2ϕ(∂P∂x)5+452α6tanϕ(∂P∂x)6+3512α7(∂P∂x)7,ϵ11=−60α4tan3ϕ(∂P∂x)4−2803α5tan2ϕ(∂P∂x)5−4159α6tanϕ(∂P∂x)6−709α7(∂P∂x)7,ϵ12=1403α5tan2ϕ(∂P∂x)5+1403α6tanϕ(∂P∂x)6+353α7(∂P∂x)7,ϵ13=−563α6tanϕ(∂P∂x)6−283α7(∂P∂x)7,ϵ14=289α7(∂P∂x)7,σ8=α∂P∂z−6α2(∂P∂z)2+556α3(∂P∂z)3−132α4(∂P∂z)4+11948α5(∂P∂z)5−12α6(∂P∂z)6+7144α7(∂P∂z)7,σ9=12α2(∂P∂z)2−40α3(∂P∂z)3+43α4(∂P∂z)4−653α5(∂P∂z)5+9718α6(∂P∂z)6−712α7(∂P∂z)7,σ10=40α3(∂P∂z)3−90α4(∂P∂z)4+2053α5(∂P∂z)5−452α6(∂P∂z)6+3512α7(∂P∂z)7,σ11=60α4(∂P∂z)4−2803α5(∂P∂z)5+4159α6(∂P∂z)6−709α7(∂P∂z)7,σ12=1403α5(∂P∂z)5−1403α6(∂P∂z)6+353α7(∂P∂z)7,σ13=563α6(∂P∂z)6−283α7(∂P∂z)7,σ14=289α7(∂P∂z)7

are constant coefficients in (65) and (66).

### 4.5. Velocity Profiles

#### 4.5.1. Velocity Profile in *x*-Direction

Equations (45), (51), (58), and (65) give the ADM solution for the velocity profile up to order three in the transverse plane:

(68) u=−ytanϕ+12(α∂P∂x+βϵ0+β2ϵ3+β3ϵ8)(y2−y)+16(βϵ1+β2ϵ4+β3ϵ9)(y3−y)+112(βϵ2+β2ϵ5+β3ϵ10)(y4−y)+120(β2ϵ6+β3ϵ11)(y5−y)+130(β2ϵ7+β3ϵ12)(y6−y)+β3ϵ1342(y7−y)+β3ϵ1456(y8−y).

#### 4.5.2. Velocity Profile in *z*-Direction

Equations (46), (52), (59), and (66) give the ADM solution for the velocity profile up to order three in the down channel direction:

(69) w=y+12(α∂P∂z+βσ0+β2σ3+β3σ8)(y2−y)+16(βσ1+β2σ4+β3σ9)(y3−y)+112(βσ2+β2σ5+β3σ10)(y4−y)+120(β2σ6+β3σ11)(y5−y)+130(β2σ7+β3σ12)(y6−y)+β3σ1342(y7−y)+β3σ1456(y8−y).

#### 4.5.3. Velocity in the Direction of the Axis of Screw

The velocity in the direction of the axis of the screw at any depth in the channel can be computed from (68) and (69) as

(70) s=wsinϕ+ucos⁡ϕ,

(71) s=12{(α∂P∂z+βσ0+β2σ3+β3σ8)sinϕ +(α∂P∂z+βϵ0+β2ϵ3+β3ϵ8)cos⁡ϕ}(y2−y)+16{(βσ1+β2σ4+β3σ9)sinϕ   +(βϵ1+β2ϵ4+β3ϵ9)cos⁡ϕ}(y3−y)+112{(βσ2+β2σ5+β3σ10)sinϕ    +(βϵ2+β2ϵ5+β3ϵ10)cos⁡ϕ}(y4−y)+120{(β2σ6+β3σ11)sinϕ    +(β2ϵ6+β3ϵ11)cos⁡ϕ}(y5−y)+130{(β2σ7+β3σ12)sinϕ    +(β2ϵ7+β3ϵ12)cos⁡ϕ}(y6−y)+β342(σ13sinϕ+ϵ13cos⁡ϕ)(y7−y)+β356(σ14sinϕ+ϵ14cos⁡ϕ)(y8−y).

Equation (71) represents the resultant velocity of the flow and shows that the forward velocity at a point in the channel only depends on the pressure gradients ∂*P*/∂*x* and ∂*P*/∂*z*.

### 4.6. Shear Stresses

Using (68)-(69) in (9) we obtain

(72) Sxy∗=Syx∗=[−tanϕ+12(−1+2y) ×(α∂P∂x+βϵ0+β2ϵ3+β3ϵ8) +16(−1+3y2)(βϵ1+β2ϵ4+β3ϵ9) +112(−1+4y3)(βϵ2+β2ϵ5+β3ϵ10)  +120(−1+5y4)(β2ϵ6+β3ϵ11) +130(−1+6y5)(β2ϵ7+β3ϵ12) +β3ϵ1342(−1+7y6)+β3ϵ1456(−1+8y7)]+hμβW×sinh⁡−1[Whα{−tanϕ+12(−1+2y)        ×(α∂P∂x+βϵ0+β2ϵ3+β3ϵ8)        +16(−1+3y2)(βϵ1+β2ϵ4+β3ϵ9)        +112(−1+4y3)(βϵ2+β2ϵ5+β3ϵ10)        +120(−1+5y4)(β2ϵ6+β3ϵ11)        +130(−1+6y5)(β2ϵ7+β3ϵ12)        +β3ϵ1342(−1+7y6)        +β3ϵ1456(−1+8y7)}],Syz∗=Szy∗=[1+12(−1+2y)(α∂P∂z+βσ0+β2σ3+β3σ8) +16(−1+3y2)(βσ1+β2σ4+β3σ9) +112(−1+4y3)(βσ2+β2σ5+β3σ10) +120(−1+5y4)(β2σ6+β3σ11) +130(−1+6y5)(β2σ7+β3σ12) +β3σ1342(−1+7y6)+β3σ1456(−1+8y7)]+hμβW×sinh⁡−1[Whα{1+12(−1+2y)        ×(α∂P∂z+βσ0+β2σ3+β3σ8)        +16(−1+3y2)(βσ1+β2σ4+β3σ9)        +112(−1+4y3)        ×(βσ2+β2σ5+β3σ10)        +120(−1+5y4)(β2σ6+β3σ11)        +130(−1+6y5)(β2σ7+β3σ12)        +β3σ1342(−1+7y6)        +β3σ1456(−1+8y7)}],

where *S*_ij_* = *S*_*ij*_/(*μW*/*h*) is the nondimensional shear stress.

The shears exerted by the fluid on the wall at *y* = 1 are

(73) Swx∗=−[−tanϕ+12(α∂P∂x+βϵ0         +β2ϵ3+β3ϵ8)  +13(βϵ1+β2ϵ4+β3ϵ9)  +14(βϵ2+β2ϵ5+β3ϵ10)  +15(β2ϵ6+β3ϵ11)   +16(β2ϵ7+β3ϵ12)+β3ϵ137+β3ϵ148]−hμβW×sinh⁡−1[Whα{−tanϕ+12(α∂P∂x+βϵ0                +β2ϵ3+β3ϵ8)          +13(βϵ1+β2ϵ4+β3ϵ9)          +14(βϵ2+β2ϵ5+β3ϵ10)          +15(β2ϵ6+β3ϵ11)+16(β2ϵ7+β3ϵ12)          +β3ϵ137+β3ϵ148}],

(74) Swz∗=−[1+12(α∂P∂z+βσ0+β2σ3+β3σ8)  +13(βσ1+β2σ4+β3σ9)  +14(βσ2+β2σ5+β3σ10)  +15(β2σ6+β3σ11)  +16(β2σ7+β3σ12)+β3σ137+β3σ148]−hμβW×sinh⁡−1[Whα{1+12(α∂P∂z+βσ0+β2σ3+β3σ8)          +13(βσ1+β2σ4+β3σ9)          +14(βσ2+β2σ5+β3σ10)          +15(β2σ6+β3σ11)          +16(β2σ7+β3σ12)          +β3σ137+β3σ148}],

where *S*_*w*_x__* = *S*_*w*_*x*__/(*μW*/*h*) and *S*_*w*_z__* = *S*_*w*_*z*__/(*μW*/*h*) are nondimensional shears at wall.

The shear force per unit width required to move the upper plate in *x*-direction is

(75) $$\begin{array}{c} \frac{F_{x}}{B}=-\int_{0}^{q_{1}}S_{w_{x}}dx; \end{array}$$

equation (75) gives

(76) $$\begin{array}{c} F_{x}^{*}=-S_{w_{x}}^{*}\delta_{1}; \end{array}$$

The shear force per unit width required to move the upper plate in *z*-direction is

(77) $$\begin{array}{c} \frac{F_{z}}{B}=-\int_{0}^{q_{2}}S_{w_{z}}dz, \end{array}$$

equation (77) gives

(78) $$\begin{array}{c} F_{z}^{*}=-S_{w_{z}}^{*}\delta_{2}, \end{array}$$

where *F*_x_* = *F*_*x*_/*μW* *B*, *F*_z_* = *F*_*z*_/*μW* *B* are dimensionless shear forces and *δ*_1_ = *q*_1_/*h*, *δ*_2_ = *q*_2_/*h* are dimensionless lengths and *q*_1_ and *q*_2_ are lengths of the channel in *x* and *z*-directions. The net shear force per unit width in the direction of the axis of the screw can be computed from (76) and (78) as

(79) $$\begin{array}{c} F^{*}=F_{z}^{*}\sin\phi +F_{x}^{*}\cos\phi , \end{array}$$

where F* = *F*/*μW* *B* is dimensionless shear force.

### 4.7. Volume Flow Rates

Volume flow rate in *x*-direction per unit width is

(80) $$\begin{array}{c} Q_{x}^{*}=\int_{0}^{1}udy, \end{array}$$

where *Q*_x_* = *Q*_*x*_/*WhB* is dimensionless volume flow rate in *x*-direction; using (68), (80) gives

(81) Qx∗=−12tanϕ−112(α∂P∂x+βϵ0+β2ϵ3+β3ϵ8)−124(βϵ1+β2ϵ4+β3ϵ9)−140(βϵ2+β2ϵ5+β3ϵ10)−160(β2ϵ6+β3ϵ11)−184(β2ϵ7+β3ϵ12)−β3ϵ13112−β3ϵ14144.

Volume flow rate in *z*-direction per unit width is

(82) $$\begin{array}{c} Q_{z}^{*}=\int_{0}^{1}wdy, \end{array}$$

where *Q*_z_* = *Q*_*z*_/*WhB* is the dimensionless volume flow rate in *z*-direction; using (69), (82) gives

(83) Qz∗=12−112(α∂P∂z+βσ0+β2σ3+β3σ8)−124(βσ1+β2σ4+β3σ9)−140(βσ2+β2σ5+β3σ10)−160(β2σ6+β3σ11)−184(β2σ7+β3σ12)−β3σ13112−β3σ14144.

Equation (71) gives the resultant volume flow rate forward in the screw channel, which is the product of the velocity and cross-sectional area integrated from the root of the screw to the barrel surface. Consider

(84) $$\begin{array}{c} Q^{*}=\frac{n}{\sin\phi }\int_{0}^{1}sdy, \end{array}$$

where Q* = *Q*/*WhB* is dimensionless volume flow rate in the direction of the axis of the screw and *n* is the number of parallel flights in a multiflight screw. Using (71), (84) gives

(85) Q∗=nsinϕ[−112{(α∂P∂z+βσ0+β2σ3+β3σ8)sinϕ       +(α∂P∂x+βϵ0+β2ϵ3+β3ϵ8)cos⁡ϕ}   −124{(βσ1+β2σ4+β3σ9)sinϕ      +(βϵ1+β2ϵ4+β3ϵ9)cos⁡ϕ}   −140{(βσ2+β2σ5+β3σ10)sinϕ      +(βϵ2+β2ϵ5+β3ϵ10)cos⁡ϕ}   −160{(β2σ6+β3σ11)sinϕ      +(β2ϵ6+β3ϵ11)cos⁡ϕ}   −184{(β2σ7+β3σ12)sinϕ      +(β2ϵ7+β3ϵ12)cos⁡ϕ}   −β3112(σ13sinϕ+ϵ13cos⁡ϕ)   −β3144(σ14sinϕ+ϵ14cos⁡ϕ)];

equation (85) can be written as

(86) $$\begin{array}{c} Q^{*}=\frac{n}{\sin\phi }\left\{Q_{z}^{*}\sin\phi +Q_{x}^{*}\cos\phi \right\}. \end{array}$$

### 4.8. Average Velocity

The average velocity in the direction of the axis of the screw is

(87) $$\begin{array}{c} \bar{s^{*}}=n\int_{0}^{1}sdy, \end{array}$$

where $\bar{s^{*}}=\overset{-}{s}/W$ is nondimensional average velocity. Using (71) in (87), we get

(88) s∗¯=n[−112{(α∂P∂z+βσ0+β2σ3+β3σ8)sinϕ     +(α∂P∂x+βϵ0+β2ϵ3+β3ϵ8)cos⁡ϕ} −124{(βσ1+β2σ4+β3σ9)sinϕ     +(βϵ1+β2ϵ4+β3ϵ9)cos⁡ϕ} −140{(βσ2+β2σ5+β3σ10)sinϕ     +(βϵ2+β2ϵ5+β3ϵ10)cos⁡ϕ}  −160{(β2σ6+β3σ11)sinϕ      +(β2ϵ6+β3ϵ11)cos⁡ϕ}  −184{(β2ϵ7+β3ϵ12)sinϕ      +(β2ϵ7+β3ϵ12)cos⁡ϕ}  −β3112(σ13sinϕ+ϵ13cos⁡ϕ)  −β3144(σ14sinϕ+ϵ14cos⁡ϕ)].

## 5. Results and Discussion

**Figure 2:**
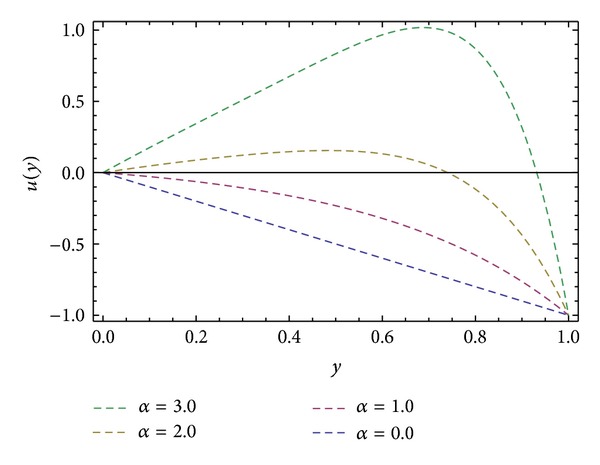
Velocity profile *u*(*y*) for different values of *α*, keeping ∂*P*/∂*x* = −0.5, *β* = 1.0, and *ϕ* = 45°.

**Figure 3:**
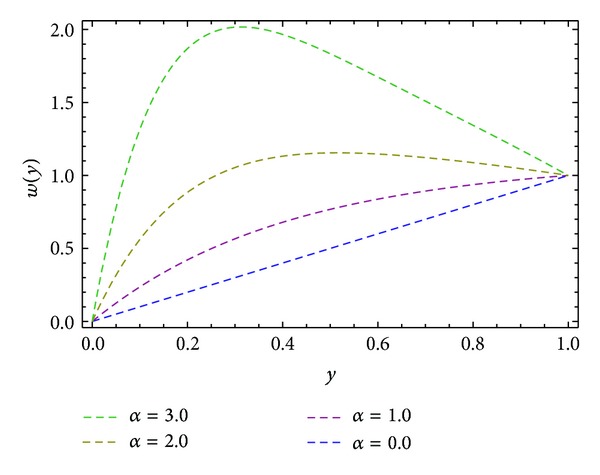
Velocity profile *w*(*y*) for different values of *α*, keeping ∂*P*/∂*z* = −0.5, *β* = 1.0, and *ϕ* = 45°.

**Figure 4:**
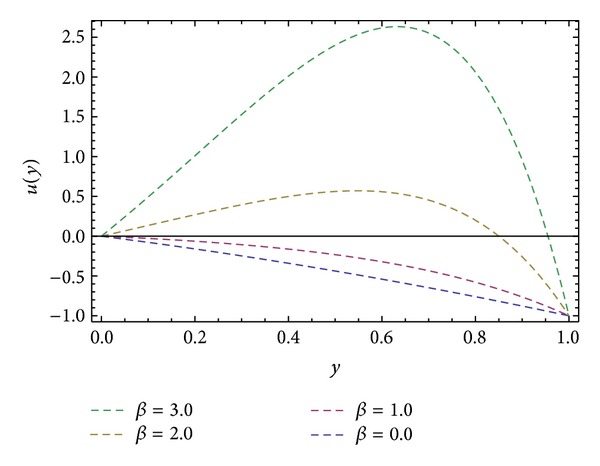
Velocity profile *u*(*y*) for different values of *β*, keeping ∂*P*/∂*x* = −0.5, *α* = 1.0, and *ϕ* = 45°.

**Figure 5:**
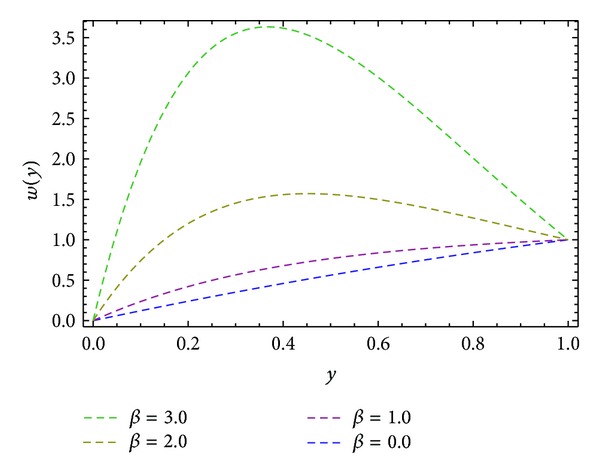
Velocity profile *w*(*y*) for different values of *β*, keeping ∂*P*/∂*z* = −0.5, *α* = 1.0, and *ϕ* = 45°.

In the present work we have considered the steady flow of an incompressible, isothermal, and homogeneous Eyring-Powell fluid in HSR (see Figure 1). By using ADM, solutions are obtained for velocity profiles in *x*- and *z*-directions and also in the direction of the axis of the screw *s*. Expressions for the shear stresses (*S*_*xy*_ and *S*_*yz*_), shear stresses at barrel surface, forces exerted on fluid, volume flow rates, and average velocity are also calculated. Here we discussed the effect of non-Newtonian parameters *α*, *β* flight angle *ϕ*, and pressure gradients ∂*P*/∂*x* and ∂*P*/∂*z* on the velocity profiles with the help of graphical representation. From Figures 2, 3, and 8, we can observe the behavior of velocity profiles against *α*.  Figure 2 is sketched for *u*, back flow is seen toward the barrel surface after some points in the channel height which show that the fluid circulates inside the confined channel; thus the velocity in *x*-direction helps in the process of mixing during processing. In Figure 3 we observe that with the increase in value of *α* the velocity *w* increases and helps to move the fluid in the forward direction in the channel. The resultant velocity *s* is shown in Figure 8, which resembles the Poiseuille flow in the channel. Due to *s* the fluid moves toward the die. It is worthwhile to note that the shear thinning occurs with the increase in value of *α*. The velocity profiles for the Newtonian case are retrieved for *α* = 0 [6]. Figures 4, 5, and 9 are plotted to notice the effect of *β* on velocities *u*, *w*, and *s*. It is seen that velocity profiles are in the same pattern for *α* and *β*. It is also noticed that both non-Newtonian parameters depict the shear thinning effects in the fluid. However, graphical representation shows that shear thinning effects of *β* are larger than *α*, as the increase in velocity profiles is observed to be larger for *β*. It is noticed that during the extrusion process, thinning/thickening of the fluid can be controlled with the proper choice of *α* and *β*. Thus, both the parameters play a vital role in the process of processing.

**Figure 6:**
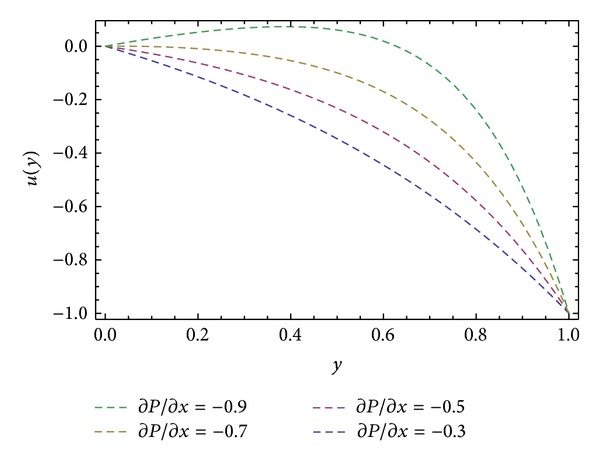
Velocity profile *u*(*y*) for different values of ∂*P*/∂*x*, keeping *α* = 1.0, *β* = 1.0, and *ϕ* = 45°.

**Figure 7:**
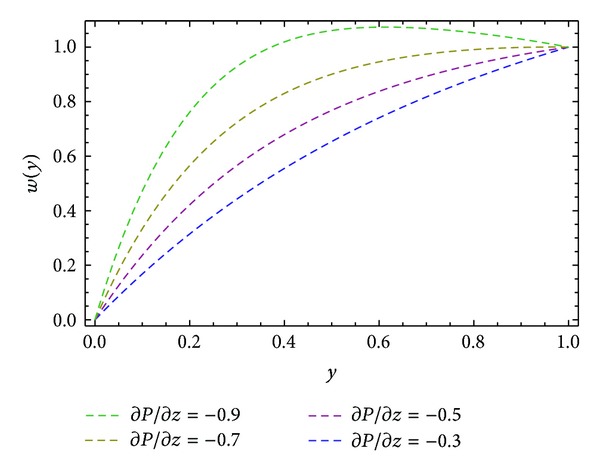
Velocity profile *w*(*y*) for different values of ∂*P*/∂*z*, keeping *α* = 1.0, *β* = 1.0, and *ϕ* = 45°.

**Figure 8:**
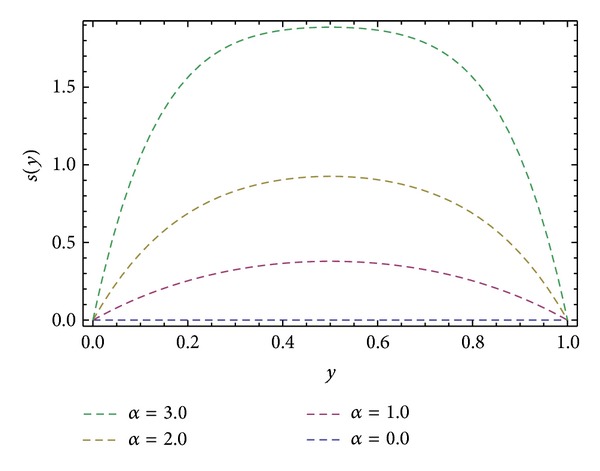
Velocity profile *s*(*y*) for different values of *α*, keeping *β* = 1.0, ∂*P*/∂*x* = −0.5, ∂*P*/∂*z* = −0.5, and *ϕ* = 45°.

**Figure 9:**
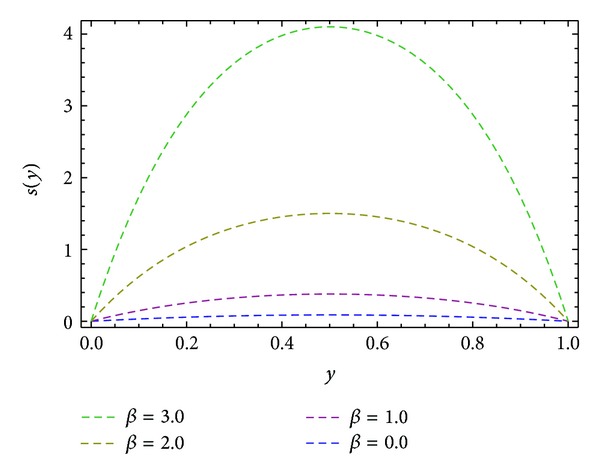
Velocity profile *s*(*y*) for different values of *β*, keeping *α* = 1.0, ∂*P*/∂*x* = −0.5, ∂*P*/∂*z* = −0.5, and *ϕ* = 45°.

**Figure 10:**
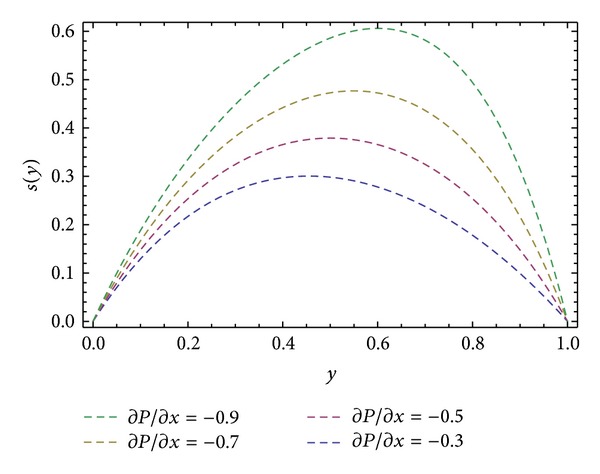
Velocity profile *s*(*y*) for different values of ∂*P*/∂*x*, keeping *α* = 1.0, *β* = 1.0, ∂*P*/∂*z* = −0.5, and *ϕ* = 45°.

**Figure 11:**
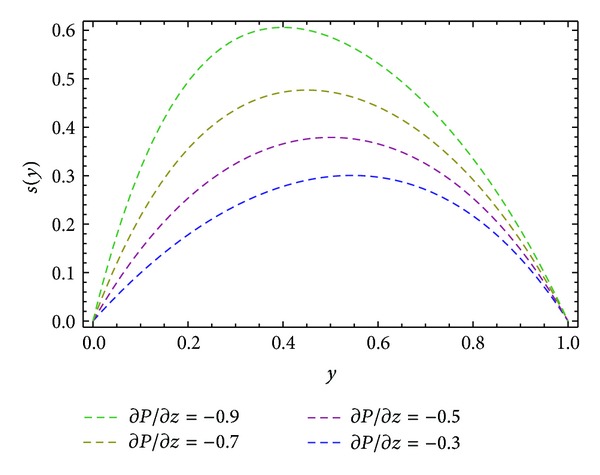
Velocity profile *s*(*y*) for different values of ∂*P*/∂*z*, keeping *α* = 1.0, *β* = 1.0, ∂*P*/∂*x* = −0.5, and *ϕ* = 45°.

**Figure 12:**
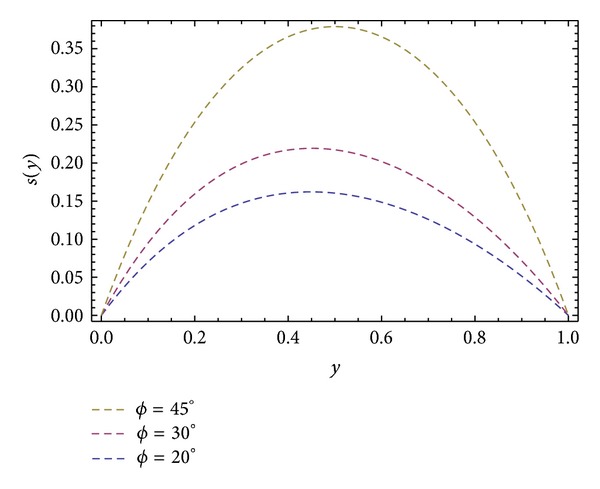
Velocity profile *s*(*y*) for different values of *ϕ*, keeping *α* = 1.0, *β* = 1.0, ∂*P*/∂*x* = −0.5, and ∂*P*/∂*z* = −0.5.

Moreover, Figures 6 and 10 are sketched for the velocity profiles *u* and *s* for different values of ∂*P*/∂*x*. It is found that the rise in pressure gradient increases speed of flow. Figures 7 and 11 are plotted for the velocity profiles *w* and *s* for different values of ∂*P*/∂*z*. It is seen that the increase in the value of ∂*P*/∂*z* increases speed of flow.  Figure 12 is plotted for different values of *ϕ*. It is observed that the resultant velocity attains its maximum value at *ϕ* = 45°, which confirms the results given in [25]. The resultant velocity given in (71), reduces to the velocity profile in *x*-direction when we take *ϕ* = 0°. When we put *ϕ* = 90° the resultant velocity given in (71) recovers the velocity profile in *z*-direction.

## 6. Conclusion

The steady flow of an isothermal, homogeneous, and incompressible Eyring-Powell fluid is investigated in HSR. Using ADM, the expressions for the velocity profiles are calculated. Expressions for the shear stresses, shear stresses at barrel surface, shear forces exerted on the fluid, volume flow rates, and average velocity of the fluid are also calculated. Graphical representation is given for the velocity profiles. It is observed that the velocity field depends on the involved parameters. The increase in the value of non-Newtonian parameters and pressure gradients increases the flow of the fluid. It can be seen that the shear thinning effect of *β* is larger than *α* in the fluid. It is also observed that the net velocity of the fluid is due to the pressure gradient. It is also noticed that the resultant velocity attains its maximum value at *ϕ* = 45°. Thus, the profound conclusion is that the extrusion process depends on the involved parameters.
